# Neighbourhood characteristics and the treated incidence rate of borderline personality pathology among young people

**DOI:** 10.1177/00048674231157274

**Published:** 2023-03-02

**Authors:** Brian O’Donoghue, Chantal Michel, Katherine N Thompson, Marialuisa Cavelti, Scott Eaton, Jennifer K Betts, Claire Fowler, Stefan Luebbers, Michael Kaess, Andrew M Chanen

**Affiliations:** 1 Department of Psychiatry, University College Dublin, Dublin, Ireland; 2 Department of Psychiatry, Royal College of Surgeons in Ireland, Dublin, Ireland; 3 Centre for Youth Mental Health, The University of Melbourne, Parkville, VIC, Australia; 4 Orygen, Parkville, VIC, Australia; 5 University Hospital of Child and Adolescent Psychiatry and Psychotherapy, University of Bern, Bern, Switzerland; 6 Centre for Forensic Behavioural Science, Swinburne University of Technology, Alphington, VIC, Australia; 7 Clinic for Child and Adolescent Psychiatry, Centre for Psychosocial Medicine, University of Heidelberg, Heidelberg, Germany

**Keywords:** Borderline personality disorder, deprivation, socioeconomic status, social fragmentation

## Abstract

**Objective:**

The impact of the wider social environment, such as neighbourhood characteristics, has not been examined in the development of borderline personality disorder. This study aimed to determine whether the treated incidence rate of full-threshold borderline personality disorder and sub-threshold borderline personality disorder, collectively termed borderline personality pathology, was associated with the specific neighbourhood characteristics of social deprivation and social fragmentation.

**Method:**

This study included young people, aged 15–24 years, who attended Orygen’s Helping Young People Early programme, a specialist early intervention service for young people with borderline personality pathology, from 1 August 2000–1 February 2008. Diagnoses were confirmed using the Structured Clinical Interview for *DSM*-IV Personality Disorders, and census data from 2006 were used to determine the at-risk population and to obtain measures of social deprivation and fragmentation.

**Results:**

The study included 282 young people, of these 78.0% (*n* = 220) were female and the mean age was 18.3 years (SD = ±2.7). A total of 42.9% (*n* = 121) met criteria for full-threshold borderline personality disorder, and 57.1% (*n* = 161) had sub-threshold borderline personality disorder, defined as having three or four of the nine *Diagnostic and Statistical Manual of Mental Disorders* (4th ed.; *DSM*-IV) borderline personality disorder criteria. There was more than a sixfold increase in the treated incidence rate of borderline personality pathology in the neighbourhoods of above average deprivation (Quartile 3) (incidence rate ratio = 6.45, 95% confidence interval: [4.62, 8.98], *p* < 0.001), and this was consistent in the borderline personality disorder sub-groups. This association was also present in the most socially deprived neighbourhood (Quartile 4) (incidence rate ratio = 1.63, 95% confidence interval: [1.10, 2.44]), however, only for those with sub-threshold borderline personality disorder. The treated incidence of borderline personality pathology increased incrementally with the level of social fragmentation (Quartile 3: incidence rate ratio = 1.93, 95% confidence interval: [1.37, 2.72], Quartile 4: incidence rate ratio = 2.38, 95% confidence interval: [1.77, 3.21]).

**Conclusion:**

Borderline personality pathology has a higher treated incidence in the more socially deprived and fragmented neighbourhoods. These findings have implications for funding and location of clinical services for young people with borderline personality pathology. Prospective, longitudinal studies should examine neighbourhood characteristics as potential aetiological factors for borderline personality pathology.

## Introduction

Borderline personality disorder (BPD) is a severe mental disorder that has its clinical onset during adolescence and young adulthood (young people). Among young people, BPD is associated with high levels of current and future morbidity, premature mortality and ranks in the top 10 contributors to burden of disease among all mental disorders (Chanen et al., 2017; Global Burden of Disease 2019 Mental Disorders Collaborators, 2022). BPD in young people is also associated with extensive social problems, including vocational disengagement (Hastrup et al., 2019; Juurlink et al., 2022), being a victim or perpetrator of interpersonal violence and non-violent offences, and family violence (Cavelti et al., 2021, 2022). Furthermore, it has the lowest published quality of life scores among any disease group (Chanen et al., 2022). Importantly, most of these problems are evident even among young people with sub-threshold BPD features, defined as having three or four *DSM*-IV/*DSM*-5 criteria (Thompson et al., 2019, 2020). Any borderline features as early as age 12 years predicts poorer outcomes in the transition to adulthood, over and above other behavioural and emotional problems (Wertz et al., 2020).

Identifying factors associated with early-stage BPD is a key component of early intervention, as it assists in identifying those at higher risk and might also improve understanding about the aetiology and treatment of the disorder. Current developmental theories identify genetic, biological and psychological vulnerabilities for the development of BPD in areas such as self and identity development, emotion regulation, and social cognition (Winsper, 2018). Evidence also supports reciprocal or mediational associations between environmental risk factors, such as early maternal bonding impairment (Fleck et al., 2021), harsh or insensitive parenting (Reinelt et al., 2014; Stepp et al., 2014), physical maltreatment and/or maternal negative expressed emotion (Belsky et al., 2012), and being a victim of bullying (Winsper et al., 2017).

However, the impact of the wider social environment on the development of BPD has been neglected, despite it being researched in other mental health disorders, primarily in psychotic disorders. For example, the incidence of psychotic disorders exhibits significant geographical variation according to neighbourhood characteristics, with much higher incidence rates in neighbourhoods with greater social deprivation, (characterised by higher levels of disadvantage, unemployment and lower average income levels) (O’Donoghue et al., 2016b). Higher rates of psychotic disorders have also been observed in neighbourhoods with higher levels of social mobility and transient populations (which is termed social fragmentation) (Ku et al., 2021), in more densely populated neighbourhoods (Kelly et al., 2010) and neighbourhoods with lower social capital (which are characterised by a weaker sense of community and trust among residence) (Rotenberg et al., 2020). While neighbourhood characteristics have been examined predominantly in psychotic disorders, there have been inconsistent findings with other mental health disorders, such as depression (Richardson et al., 2015). Within a cohort of individuals attending an early intervention for psychosis service, it was found that the presence of a concurrent diagnosis of a personality disorder was not associated with the level of neighbourhood social deprivation (Ban et al., 2020).

This established association between the incidence of psychotic disorders and neighbourhood characteristics has enabled service planners to reliably predict the expected incidence of psychotic disorders in different regions and resource early intervention for psychosis services accordingly (Kirkbride et al., 2013). In addition to having immediate clinical applications, it has also led researchers to investigate whether there are factors in these neighbourhoods which might contribute to the development of the disorder, such as a lack of green spaces (Engemann et al., 2020), air pollution (Newbury et al., 2021) or loneliness (Lim et al., 2018). Interestingly, some studies found gender differences. For example, the incidence of psychotic disorders in females was greatly increased in neighbourhoods that were more socially fragmented (Omer et al., 2014) or had lower social capital (O’Donoghue et al., 2016a). This raises the possibility that females might be more susceptible to the effects of the wider neighbourhood environment in relation to the development of mental health disorders.

Only one study has examined the associations between neighbourhood characteristics and BPD, and this was conducted among adults with late-stage disorder (Walsh et al., 2013). This found that neighbourhood socioeconomic status (SES) was associated with more symptoms and lower functioning, even after adjusting for individual-level SES. This study did not examine gender effects, despite BPD being more common among females in clinical epidemiological samples (Skodol and Bender, 2003).

The association between the incidence of BPD and neighbourhood factors warrants further investigation to improve understanding of the role of environmental factors in the aetiology of BPD. Findings would also assist in planning future service provision, development of prevention programmes and allocation of healthcare resources. Therefore, this study aimed to determine whether the treated incidence rate of full-threshold BPD and/or sub-threshold BPD (collectively termed borderline personality pathology [BPP]) is associated with the neighbourhood characteristics of social deprivation and social fragmentation.

## Method

### Design

This was a naturalistic, prospective cohort study that included young people who attended a specialist programme for BPP during the study period.

### Setting

This study was conducted at Orygen’s Helping Young People Early (HYPE) programme, a specialist, early intervention service for young people with BPP (Chanen et al., 2009). Orygen is the government-funded specialist youth mental health service for young people residing within the north-western and western regions of metropolitan Melbourne, Australia. At the time of recruitment, Orygen’s catchment population exceeded 1 million residents, of whom approximately 150,000 were aged between 15 and 24 years.

### Participants

Participants were aged 15–24 years, who met at least three of the nine *DSM*-IV BPD criteria and who received treatment at HYPE between August 2000 and February 2008. There were no exclusion criteria, and the sample included young people with concurrent substance abuse, suicidal ideation or self-harming behaviours or antisocial behaviour.

### Demographic and clinical data

Demographic and diagnostic information were obtained at the time of entry to the service and were sourced from client records, collected as part of routine clinical practice. Gender, age, education, residential postcode and occupation were collated. Diagnostic data were assessed using the Structured Clinical Interview for *DSM*-IV Personality Disorders (SCID-II) (First et al., 1997). Co-occurring mental state diagnoses were assessed in accordance with *DSM*-IV (First et al., 1995). They were categorised into depressive disorders (including major depressive disorder, dysthymic disorder), anxiety disorders (including generalised anxiety disorder, panic disorder, obsessive-compulsive disorder, posttraumatic stress disorder and acute stress disorder), substance use disorders (including substance abuse and dependence, excluding nicotine) and other disorders (e.g. psychotic disorders, bipolar disorders, attention-deficit and disruptive behaviour disorders) (Table 1).

**Table 1.** Demographic and clinical characteristics of total cohort and those with full-threshold BPD and sub-threshold BPD.

Baseline characteristics	Total BPP cohort *n* = 282	Full-threshold BPD *n* = 121	Sub-threshold BPD *n* = 161	*p*
	Means (SDs)/ percentages (*n*)	Means (SDs)/ percentages (*n*)	Means (SDs)/ percentages (*n*)	
Demographics				
Age at presentation, years	18.3 (2.7)	18.4 (2.7)	18.2 (2.6)	0.489
Sex: % female	78.0 (220)	78.5 (95)	77.6 (125)	0.861
Occupation – % student/working	64.8 (147)	65.3 (64)	64.3 (83)	0.880
Illness and clinical features				
Concurrent *DSM*-IV diagnosis, %				
Depression	65.7 (184)	67.5 (81)	64.4 (103)	0.586
Anxiety disorder	28.6 (80)	25.0 (30)	31.3 (50)	0.252
Substance abuse/dependence	20.4 (57)	20.0 (24)	20.6 (33)	0.898
Other disorders	31.2 (88)	34.7 (42)	28.6 (46)	0.265
Borderline personality pathology				
Abandonment	22.7 (64)	16.5 (20)	27.3 (44)	0.032
Relationship instability	48.2 (136)	47.9 (58)	48.4 (78)	0.932
Identity disturbance	34.4 (97)	30.6 (37)	37.3 (60)	0.242
Impulsivity	36.9 (104)	38.8 (47)	35.4 (57)	0.554
Suicidality/self-harm	64.5 (182)	61.2 (74)	67.1 (108)	0.303
Affective instability	66.7 (188)	68.6 (83)	65.2 (105)	0.551
Chronic feelings of emptiness	45.4 (128)	43.8 (53)	46.6 (75)	0.642
Inappropriate anger	67.0 (189)	67.8 (82)	66.5 (107)	0.817
Dissociation/paranoid ideation	15.3 (43)	14.2 (17)	16.1 (26)	0.648

BPP: borderline personality pathology; BPD: borderline personality disorder; SD: standard deviation.

### Classification of neighbourhood characteristics

The level of social deprivation at the postcode level was determined from the 2006 census data available from the Australian Bureau of Statistics. The specific measure of deprivation was the ‘the Index of Relative Socio-economic Advantage and Disadvantage’ within the Socio-Economic Index for Areas (SEIFA) (www.abs.gov.au). A score for each postcode was created and was standardised against a mean of 1000 with a standard deviation of 100. Each postcode was assigned to a quartile. The most affluent neighbourhoods were in Quartile 1, neighbourhoods of above average affluence (but not the most affluent) were in Quartile 2, neighbourhoods of above average social deprivation (but not the most deprived) were in Quartile 3, and the most socially deprived neighbourhoods were in Quartile 4.

Social fragmentation referred to the mobility and transitory nature of the community and was a composite measure comprised of four census variables: the percentage of single-person households, dwellings rented, persons having lived at a different address 1-year prior and (socially defined) unmarried persons (Congdon, 1996). Data for these variables were collated for all 665 postcodes in the Australian state of Victoria, 3 of which were excluded due to zero values. Sample mean and standard deviation were calculated for each of the four census variables within the remaining population of 662 postcodes. For each postcode, the deviation from the mean (*z*-score) was calculated for each of these census variables. The social fragmentation score for a postcode was calculated by adding the postcode’s *z*-scores for each of these four census variables. Each postcode was assigned to a quartile. Neighbourhoods that were the least fragmented were in Quartile 1, neighbourhoods with below average fragmentation were in Quartile 2, neighbourhoods with above average fragmentation were in Quartile 3, and the most socially fragmented neighbourhoods were in Quartile 4.

Social capital can be measured by a survey evaluating the level of trust and cohesion among residents (Kirkbride et al., 2008). Without this direct measurement, a proxy measure such as voter turnout can be used (O’Donoghue et al., 2016a). However, Australia has mandatory voting, so this neighbourhood factor was not examined in this study. In addition, there was insufficient variability in the level of population density within the catchment area for it to explain any potential variation in the incidence of BPP, and therefore, it was not examined.

### Statistical analyses

The incidence rate ratios (IRRs) were calculated using the Poisson command in Stata v. 14 (StataCorp, 2019), and sex and age were adjusted for in the model. *T*-tests were used to determine whether there was a difference in means between groups, and chi-square tests were used to determine whether there was a difference between groups in relation to categorical variables.

### Ethical approval

This study was approved by Melbourne Health HREC 2008.614, and routinely collected data from the initial clinical assessment were utilised in this study and a waiver of consent was granted, which facilitated obtaining a representative cohort.

## Results

### Description of participants

Complete data were available for a total of 282 young people. Of these, 78.0% (*n* = 220) were female, with mean age 18.3 years (SD = 2.7). A total of 42.9% (*n* = 121) met criteria for full-threshold BPD, and 57.1% (*n* = 161) met criteria for sub-threshold BPD, having either three or four *DSM*-IV BPD criteria. Table 1 shows demographic and clinical characteristics of the sample. No significant between-group differences were found, except that members of the sub-threshold BPD group were more likely to meet the ‘fear of abandonment’ diagnostic criterion.

### Social deprivation and treated incidence

There was more than a sixfold increase in the treated incidence rate of BPP in the neighbourhoods with above average deprivation (Quartile 3) (IRR = 6.45, 95% confidence interval [CI]: [4.62, 8.98], *p* < 0.001), compared with the most affluent neighbourhoods (Quartile 1), when adjusting for age and sex. This finding was consistent across the full-threshold (IRR = 4.77, 95% CI: [2.97, 7.66]) and sub-threshold (IRR = 8.37, 95% CI: [5.22, 13.40]) BPD groups. The treated incidence rate was also higher in the most socially deprived neighbourhood (Quartile 4) (IRR = 1.63, 95% CI: [1.10, 2.44]). However, this association was only present for the sub-threshold BPD group (IRR = 2.04, 95% CI: [1.17, 3.55]) (Table 2).

**Table 2.** Incidence rate ratios of the treated incidence of BPP according to social deprivation and fragmentation.

Population	Quartile	Personal years population-at-risk	Cases	Incidence rate ratio	95% confidence interval		*p*
					Lower	Upper	
Social deprivation							
All cases	Q1 (most affluent)	302,723	47	Ref			
	Q2	501,150	44	0.49	0.33	0.75	0.001
	Q3	124,770	141	6.45	4.62	8.98	<0.001
	Q4 (most deprived)	176,108	50	1.63	1.10	2.44	0.016
Full-threshold BPD	Q1 (most affluent)	302,723	25	Ref			
	Q2	501,150	21	0.44	0.25	0.79	0.006
	Q3	124,770	56	4.77	2.97	7.66	<0.001
	Q4 (most deprived)	176,108	21	1.28	0.71	2.29	0.41
Sub-threshold BPD	Q1 (most affluent)	302,723	22	Ref			
	Q2	501,150	23	0.55	0.31	1.00	0.050
	Q3	124,770	85	8.37	5.22	13.40	<0.001
	Q4 (most deprived)	176,108	29	2.04	1.17	3.55	0.012
Social fragmentation							
All cases	Q1 (lowest)	168,209	70	Ref			
	Q2	278,439	33	0.25	0.16	0.38	<0.001
	Q3	69,322	63	1.93	1.37	2.72	<0.001
	Q4 (highest)	97,720	116	2.38	1.77	3.21	<0.001
Full-threshold BPD	Q1 (lowest)	168,209	33	Ref			
	Q2	278,439	15	0.24	0.13	0.44	<0.001
	Q3	69,322	24	1.56	0.92	2.64	0.098
	Q4 (highest)	97,720	49	2.13	1.37	3.32	0.001
Sub-threshold BPD	Q1 (lowest)	168,209	37	Ref			
	Q2	278,439	18	0.26	0.15	0.45	<0.001
	Q3	69,322	39	2.26	1.44	3.55	<0.001
	Q4 (highest)	97,720	67	2.61	1.74	3.90	<0.001

BPD: borderline personality disorder.

### Social fragmentation and treated incidence

With the neighbourhoods with the lowest level of social fragmentation (Quartile 1) as a reference, the treated incidence of BPP increased incrementally from the neighbourhoods with above average social fragmentation (Quartile 3) (IRR = 1.93, 95% CI: [1.37, 2.72]) to the most socially fragmented neighbourhoods (Quartile 4) (IRR = 2.38, 95% CI: [1.77, 3.21]). These findings were consistent in the sub-threshold BPD group. The association between social fragmentation and the treated incidence of full-threshold BPD was only significant in the most socially fragmented neighbourhoods (Quartile 4) (IRR = 2.13, 95% CI: [1.37, 3.32]), as displayed in Table 2.

## Discussion

### Summary of findings

This novel study examined neighbourhood characteristics and the treated incidence rate of BPP among young people attending a specialised early intervention programme for BPP. The main findings from this study are that the treated incidence of BPP was significantly higher in both more socially deprived and more fragmented neighbourhoods.

### Clinical implications

These findings have important implications in relation to the funding and location of clinical services for young people with BPP. Historically, mental health services have been funded on a *per capita* basis. However, this is inequitable, as there is a higher incidence of mental health disorders in more deprived and socially fragmented areas. Consequently, more affluent neighbourhoods tend to be over-resourced, to the detriment of more deprived areas. This disparity is well established for people with psychotic disorders (Eaton et al., 2019). The current findings indicate that early intervention services for BPP should also be resourced according to the predicted need. This is also in keeping with the global evolution of youth mental health services (McGorry et al., 2022) designed to address severe mental health disorders during their peak period of onset in the first three decades of life (McGorry et al., 2011). The current study demonstrates that there are similarities across severe mental disorders in terms of the association between the treated incidence rate and neighbourhood factors. All of these factors lend further support to the youth mental health model, alongside appropriate specialist care when needed.

### Insights into the aetiology of BPP

The current findings might also provide insights into the aetiology of BPD. There has been a long-standing debate as to whether the higher prevalence of psychotic disorders among people of lower SES and more deprived neighbourhoods is a result of ‘social drift’ or social causation. The former is hypothesised to arise from cognitive and functional decline associated with the disorder. The latter hypothesises that the environment might increase the risk for the disorder (Kwok, 2014). The current clinical sample with BPP included young people, most of whom were still students. Therefore, it is less likely that social drift is responsible for the observed findings. The current findings suggest that the wider social environment, specifically the neighbourhood of residence, might also play a role in the aetiology of personality pathology. In relation to the potential effects of social deprivation, it is a curious finding that the increased incidence of BPP was much greater in Quartile 3, corresponding to neighbourhoods with above average social deprivation, than in Quartile 4, which had neighbourhoods with the highest level of social deprivation. There could be two potential explanations for this finding. First, it has been demonstrated that ‘relative social deprivation’ can also be harmful to health and this is thought to be explained by the negative social comparisons that individuals may make who are residing in more deprived areas but in close proximity to more affluent areas (Zhang et al., 2013). Second, these results represent the treated incidence of BPP, and this might not reflect the actual incidence of BPP. It might be the case of the inverse care law (Hart, 1971), whereby young people with BPP in the most deprived areas are not accessing the care that they need, in comparison with those in the above average deprived areas.

In relation to the findings of a higher incidence of BPP in the more socially fragmented neighbourhoods, these areas are characterised by high levels of residential mobility, which is known to increase the risk for all mental health disorders, including personality disorder (Mok et al., 2016). This might hinder a vulnerable individual from establishing important longer-term relationships immediately outside of the family. In addition, the affected individual living in the more socially fragmented neighbourhoods might have had higher residential mobility themselves (possibly due to the interpersonal dysfunction of having BPD), again resulting in disruptions and changes to schooling and friendships.

There is also a common, unexpected trend across the findings in that the treated incidence rates were consistently lower in the second quartiles, which represent above average affluence and below average social fragmentation. Therefore, while having higher relative social deprivation could be harmful to health, it could be hypothesised that that there is a protective effect for individuals residing in areas of above average affluence, and below average social fragmentation, but not the most affluent or least fragmented areas. Further research with prospective, longitudinal designs is required to definitively answer these questions, and the current findings suggest that such studies should be pursued.

### Strengths and limitations

Limitations to the study include that this was a clinical cohort, with participants offered care based upon assessment of their needs. As there can be a high demand for government-funded clinical services, if a young person had the means to access care privately then they might be referred for care external to the clinical service. This might have introduced bias into the sample by including more young people from a lower SES and from more deprived and socially fragmented neighbourhoods. However, this is unlikely because, at the time the sample was collected, there were few alternatives to Orygen. A further limitation is that postcodes were taken as the level of neighbourhood division. Some postcodes show within-postcode diversity of affluence and deprivation that is lost when these are collated into the postcode’s SEIFA measure of deprivation. Finally, data on parental SES were not collected, so this could not be adjusted for in the multiple regression analysis.

## Conclusion

BPP has a higher treated incidence in neighbourhoods that have greater relative social deprivation, particularly in those with above average deprivation, and in neighbourhoods with relatively higher social fragmentation. These findings suggest that services for BPP should be funded on the basis of need, not per capita. Moreover, social fragmentation and social deprivation should be investigated in the aetiology of BPP, using prospective, longitudinal designs.

## References

[bibr1-00048674231157274] Ban KY Osborn DPJ Hameed Y , et al. (2020) Personality disorder in an Early Intervention Psychosis cohort: Findings from the Social Epidemiology of Psychoses in East Anglia (SEPEA) study. PLoS ONE 15: e0234047. 10.1371/journal.pone.0234047 PMC7274401 32502161

[bibr2-00048674231157274] Belsky DW Caspi A Arseneault L , et al. (2012) Etiological features of borderline personality related characteristics in a birth cohort of 12-year-old children. Development and Psychopathology 24: 251–265. 22293008 10.1017/S0954579411000812 PMC3547630

[bibr3-00048674231157274] Cavelti M Thompson K Betts J , et al. (2021) Borderline personality disorder diagnosis and symptoms in outpatient youth as risk factors for criminal offenses and interpersonal violence. Journal of Personality Disorders 35: 23–37. 33779276 10.1521/pedi_2021_35_503

[bibr4-00048674231157274] Cavelti M Thompson K Betts J , et al. (2022) Young people with borderline personality disorder have an increased lifetime risk of being the victim of interpersonal violence. Journal of Interpersonal Violence 37: NP10642–NP10660. 10.1177/0886260520986270 33461382

[bibr5-00048674231157274] Chanen AM Betts JK Jackson H , et al. (2022) A comparison of adolescent versus young adult outpatients with first-presentation borderline personality disorder: Findings from the MOBY randomized controlled trial. Canadian Journal of Psychiatry / Revue Canadienne De Psychiatrie 67: 26–38. 33576244 10.1177/0706743721992677 PMC8811246

[bibr6-00048674231157274] Chanen AM McCutcheon LK Germano D , et al. (2009) The HYPE Clinic: An early intervention service for borderline personality disorder. Journal of Psychiatric Practice 15: 163–172. 19461389 10.1097/01.pra.0000351876.51098.f0

[bibr7-00048674231157274] Chanen AM Sharp C Hoffman P , et al. (2017) Prevention and early intervention for borderline personality disorder: A novel public health priority. World Psychiatry 16: 215–216. 28498598 10.1002/wps.20429 PMC5428197

[bibr8-00048674231157274] Congdon P (1996) Suicide and parasuicide in London: A small-area study. Urban Studies 33: 137–158.

[bibr9-00048674231157274] Eaton S Harrap B Downey L , et al. (2019) Incidence of treated first episode psychosis from an Australian early intervention service and its association with neighbourhood characteristics. Schizophrenia Research 209: 206–211. 31130401 10.1016/j.schres.2019.04.017

[bibr10-00048674231157274] Engemann K Pedersen CB Agerbo E , et al. (2020) Association between childhood green space, genetic liability, and the incidence of schizophrenia. Schizophrenia Bulletin 46: 1629–1637. 32415773 10.1093/schbul/sbaa058 PMC8496913

[bibr11-00048674231157274] First M Gibbon M Spitzer RL , et al. (1997) Structured Clinical Interview for DSM-IV Axis II Personality Disorders: SCID-II. Washington, DC: American Psychiatric Association.

[bibr12-00048674231157274] First M Spitzer RL Gibbon M , et al. (1995) Structured Clinical Interview for DSM-IV Axis 1 Disorders. New York: New York State Psychiatric Institute.

[bibr13-00048674231157274] Fleck L Fuchs A Moehler E , et al. (2021) Maternal bonding impairment predicts personality disorder features in adolescence: The moderating role of child temperament and sex. Personality Disorders 12: 475–483. 33570973 10.1037/per0000433

[bibr14-00048674231157274] Global Burden of Disease 2019 Mental Disorders Collaborators (2022) Global, regional, and national burden of 12 mental disorders in 204 countries and territories, 1990–2019: A systematic analysis for the Global Burden of Disease Study 2019. The Lancet Psychiatry 9: 137–150. 35026139 10.1016/S2215-0366(21)00395-3 PMC8776563

[bibr15-00048674231157274] Hart JT (1971) The inverse care law. The Lancet 1: 405–412. 10.1016/s0140-6736(71)92410-x 4100731

[bibr16-00048674231157274] Hastrup LH Kongerslev MT Simonsen E (2019) Low vocational outcome among people diagnosed with borderline personality disorder during first admission to mental health services in Denmark: A nationwide 9-year register-based study. Journal of Personality Disorders 33: 326–340. 29505387 10.1521/pedi_2018_32_344

[bibr17-00048674231157274] Juurlink TT Betts JK Nicol K , et al. (2022) Characteristics and predictors of educational and occupational disengagement among outpatient youth with borderline personality disorder. Journal of Personality Disorders 36: 116–128. 34427492 10.1521/pedi_2021_35_534

[bibr18-00048674231157274] Kelly BD O’Callaghan E Waddington JL , et al. (2010) Schizophrenia and the city: A review of literature and prospective study of psychosis and urbanicity in Ireland. Schizophrenia Research 116: 75–89. 19897342 10.1016/j.schres.2009.10.015

[bibr19-00048674231157274] Kirkbride JB Boydell J Ploubidis GB , et al. (2008) Testing the association between the incidence of schizophrenia and social capital in an urban area. Psychological Medicine 38: 1083–1094. 17988420 10.1017/S0033291707002085

[bibr20-00048674231157274] Kirkbride JB Jackson D Perez J , et al. (2013) A population-level prediction tool for the incidence of first-episode psychosis: Translational epidemiology based on cross-sectional data. BMJ Open 3: e001998. 10.1136/bmjopen-2012-001998 PMC3585967 23399458

[bibr21-00048674231157274] Ku BS Compton MT Walker EF , et al. (2021) Social fragmentation and schizophrenia: A systematic review. Journal of Clinical Psychiatry 83: 21r13941. 10.4088/JCP.21r13941 PMC13103956 34875149

[bibr22-00048674231157274] Kwok W (2014) Is there evidence that social class at birth increases risk of psychosis? A systematic review. The International Journal of Social Psychiatry 60: 801–808. 24608029 10.1177/0020764014524737

[bibr23-00048674231157274] Lim MH Gleeson JFM Alvarez-Jimenez M , et al. (2018) Loneliness in psychosis: A systematic review. Social Psychiatry and Psychiatric Epidemiology 53: 221–238. 29327166 10.1007/s00127-018-1482-5

[bibr24-00048674231157274] McGorry PD Mei C Chanen A , et al. (2022) Designing and scaling up integrated youth mental health care. World Psychiatry 21: 61–76. 35015367 10.1002/wps.20938 PMC8751571

[bibr25-00048674231157274] McGorry PD Purcell R Goldstone S , et al. (2011) Age of onset and timing of treatment for mental and substance use disorders: Implications for preventive intervention strategies and models of care. Current Opinion in Psychiatry 24: 301–306. 21532481 10.1097/YCO.0b013e3283477a09

[bibr26-00048674231157274] Mok PL Webb RT Appleby L , et al. (2016) Full spectrum of mental disorders linked with childhood residential mobility. Journal of Psychiatric Research 78: 57–64. 27074536 10.1016/j.jpsychires.2016.03.011 PMC4866579

[bibr27-00048674231157274] Newbury JB Stewart R Fisher HL , et al. (2021) Association between air pollution exposure and mental health service use among individuals with first presentations of psychotic and mood disorders: Retrospective cohort study. The British Journal of Psychiatry 219: 678–685. 35048872 10.1192/bjp.2021.119 PMC8636613

[bibr28-00048674231157274] O’Donoghue B Lyne JP Renwick L , et al. (2016a) Neighbourhood characteristics and the incidence of first-episode psychosis and duration of untreated psychosis. Psychological Medicine 46: 1367–1378. 27032697 10.1017/S003329171500286X

[bibr29-00048674231157274] O’Donoghue B Roche E Lane A (2016b) Neighbourhood level social deprivation and the risk of psychotic disorders: A systematic review. Social Psychiatry and Psychiatric Epidemiology 51: 941–950. 27178430 10.1007/s00127-016-1233-4

[bibr30-00048674231157274] Omer S Kirkbride JB Pringle DG , et al. (2014) Neighbourhood-level socio-environmental factors and incidence of first episode psychosis by place at onset in rural Ireland: The Cavan-Monaghan First Episode Psychosis Study [CAMFEPS]. Schizophrenia Research 152: 152–157. 24342585 10.1016/j.schres.2013.11.019 PMC3906531

[bibr31-00048674231157274] Reinelt E Stopsack M Aldinger M , et al. (2014) Longitudinal transmission pathways of borderline personality disorder symptoms: From mother to child? Psychopathology 47: 10–16. 23713057 10.1159/000345857

[bibr32-00048674231157274] Richardson R Westley T Gariépy G , et al. (2015) Neighborhood socioeconomic conditions and depression: A systematic review and meta-analysis. Social Psychiatry and Psychiatric Epidemiology 50: 1641–1656. 26164028 10.1007/s00127-015-1092-4

[bibr33-00048674231157274] Rotenberg M Anderson KK McKenzie K (2020) Social capital and psychosis: A scoping review. Social Psychiatry and Psychiatric Epidemiology 55: 659–671. 31802174 10.1007/s00127-019-01812-9

[bibr34-00048674231157274] Skodol AE Bender DS (2003) Why are women diagnosed borderline more than men? The Psychiatric Quarterly 74: 349–360. 14686459 10.1023/a:1026087410516

[bibr35-00048674231157274] StataCorp (2019) Stata/IC 15.1 for Windows (Version 15.1). College Station, TX: StataCorp.

[bibr36-00048674231157274] Stepp SD Whalen DJ Scott LN , et al. (2014) Reciprocal effects of parenting and borderline personality disorder symptoms in adolescent girls. Development and Psychopathology 26: 361–378. 24443951 10.1017/S0954579413001041 PMC4103652

[bibr37-00048674231157274] Thompson KN Jackson H Cavelti M , et al. (2019) The clinical significance of subthreshold borderline personality disorder features in outpatient youth. Journal of Personality Disorders 33: 71–81. 30036169 10.1521/pedi_2018_32_330

[bibr38-00048674231157274] Thompson KN Jackson H Cavelti M , et al. (2020) Number of borderline personality disorder criteria and depression predict poor functioning and quality of life in outpatient youth. Journal of Personality Disorders 34: 785–798. 30689518 10.1521/pedi_2019_33_411

[bibr39-00048674231157274] Walsh Z Shea MT Yen S , et al. (2013) Socioeconomic-status and mental health in a personality disorder sample: The importance of neighborhood factors. Journal of Personality Disorders 27: 820–831. 22984860 10.1521/pedi_2012_26_061 PMC4628287

[bibr40-00048674231157274] Wertz J Caspi A Ambler A , et al. (2020) Borderline symptoms at age 12 signal risk for poor outcomes during the transition to adulthood: Findings from a genetically sensitive longitudinal cohort study. Journal of the American Academy of Child and Adolescent Psychiatry 59: 1165–1177. 31325594 10.1016/j.jaac.2019.07.005 PMC6980181

[bibr41-00048674231157274] Winsper C (2018) The aetiology of borderline personality disorder (BPD): Contemporary theories and putative mechanisms. Current Opinion in Psychology 21: 105–110. 29182951 10.1016/j.copsyc.2017.10.005

[bibr42-00048674231157274] Winsper C Hall J Strauss VY , et al. (2017) Aetiological pathways to borderline personality disorder symptoms in early adolescence: Childhood dysregulated behaviour, maladaptive parenting and bully victimization. Borderline Personality Disorder and Emotion Dysregulation 4: 10. 28588894 10.1186/s40479-017-0060-x PMC5457614

[bibr43-00048674231157274] Zhang X Cook PA Lisboa PJ , et al. (2013) The effects of deprivation and relative deprivation on self-reported morbidity in England: An area-level ecological study. International Journal of Health Geographics 12: 5. 23360584 10.1186/1476-072X-12-5 PMC3623854

